# Skyrmion Formation in Nanodisks Using Magnetic Force Microscopy Tip

**DOI:** 10.3390/nano11102627

**Published:** 2021-10-06

**Authors:** Mateusz Zelent, Iuliia V. Vetrova, Jan Šoltýs, Xiaoguang Li, Yan Zhou, Vladislav A. Gubanov, Alexandr V. Sadovnikov, Tomas Šcepka, Jan Dérer, Roman Stoklas, Vladimír Cambel, Michal Mruczkiewicz

**Affiliations:** 1 Institute of Spintronics and Quantum Information, Faculty of Physics, Adam Mickiewicz University in Poznan, ul. Uniwersytetu Poznańskiego 2, PL-61-614 Poznan, Poland; 2 Institute of Electrical Engineering, Slovak Academy of Sciences, Dubravska Cesta 9, 841 04 Bratislava, Slovakia; Jan.Soltys@savba.sk (J.Š.); elekscep@savba.sk (T.Š.); elekder@savba.sk (J.D.); elekstok@savba.sk (R.S.); elekcamb@savba.sk (V.C.); 3 College of Engineering Physics, Shenzhen Technology University, Shenzhen 518118, China; lixiaoguang@sztu.edu.cn; 4 School of Science and Engineering, The Chinese University of Hong Kong, Shenzhen 518172, China; zhouyan@cuhk.edu.cn; 5 Laboratory “Magnetic Metamaterials”, Saratov State University, Astrakhanskaya Street 83, 410012 Saratov, Russia; vladmeen@gmail.com (V.A.G.); SadovnikovAV@gmail.com (A.V.S.); 6 Centre For Advanced Materials Application CEMEA, Slovak Academy of Sciences, Dubravska Cesta 9, 845 11 Bratislava, Slovakia

**Keywords:** skyrmions, multilayer structure, magnetic states, micromagnetic simulation, magnetic force microscopy

## Abstract

We demonstrated numerically the skyrmion formation in ultrathin nanodisks using a magnetic force microscopy tip. We found that the local magnetic field generated by the magnetic tip significantly affects the magnetization state of the nanodisks and leads to the formation of skyrmions. Experimentally, we confirmed the influence of the local field on the magnetization states of the disks. Micromagnetic simulations explain the evolution of the magnetic state during magnetic force microscopy scanning and confirm the possibility of skyrmion formation. The formation of the horseshoe magnetic domain is a key transition from random labyrinth domain states into the skyrmion state. We showed that the formation of skyrmions by the magnetic probe is a reliable and repetitive procedure. Our findings provide a simple solution for skyrmion formation in nanodisks.

## 1. Introduction

Magnetic skyrmions are circular domains surrounded by a single chirality domain wall [1,2,3]. They are characterized by small size and robustness against the external perturbations which makes them a perspective for modern memory storage devices as an information carrier [4,5]. Skyrmions can be stabilized in ultrathin films by the interplay of an external magnetic field, perpendicular anisotropy and the Dzyaloshinskii–Moriya interaction (DMI) that is induced at the interfaces of the ferromagnetic/non-magnetic metal [6]. In the case of multilayer structures, there is additional contribution from the dipolar interactions that leads to the stabilization of stray field skyrmions [7,8]. It was found that confinement due to geometry can increase the stability of the skyrmion significantly [9]. Therefore, low-dimensional patterned structures can serve as hosts of reconfigurable magnetic states stable at room temperature [10,11]. Measuring and controlling the nucleation of skyrmions in patterned geometries still remains an important task [10,12,13].

The challenge of the experimental study of skyrmions is to find an efficient technique for forming stable skyrmions. Recently, individual skyrmion manipulation in a Pt/CoFeB/MgO multilayer using a magnetic force microscopy (MFM) tip was reported [14]. This method was also used for the nucleation of domains, skyrmions and skyrmion lattices in various non-patterned ultrathin films [15,16,17,18]. The effect of the magnetization reversal of skyrmions by the MFM tip was studied in symmetric Pt/Co/Pt thin films consisting of modified cylindrical regions with a diameter of 100 nm [19]. Highly metastable hedgehog skyrmions in soft magnetic permalloy nanodots with a diameter of 70 nm and height of 30 nm were presented in Reference [20].

In this study, we demonstrated experimentally the switching of domain states to a single-domain state by a local field induced by an MFM tip. The numerical simulations showed a possible evolution of the domain state to a single-domain state. Further, induction of the skyrmions from the domain state was demonstrated by numerical simulations when the pinning centers were introduced at the boundary of the dot.

## 2. Materials and Methods

### 2.1. Fabrication

**Figure 1:**
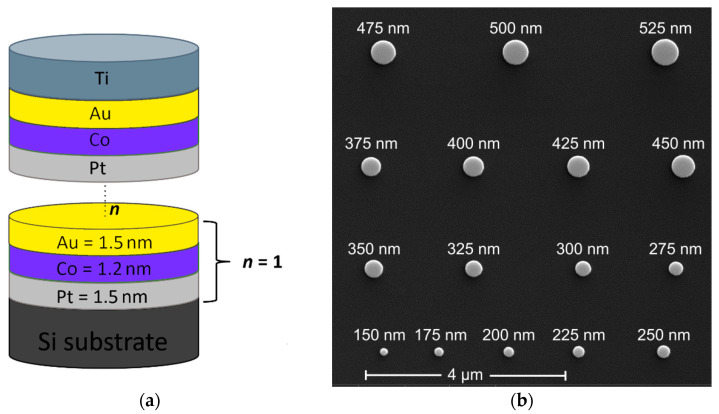
(**a**) Schematic representation of multilayer nanodot based on ultrathin Co layers placed between two different heavy metals (Au, Pt), where n represents the number of trilayer Au/Co/Pt unit-cell repeats. The value of n ranges from 4 to 7. (**b**) Scanning electron microscope top view of an array of multilayer dots patterned by the electron-beam lithography and etching method. The 16 nanodots have diameters in the range of 150–525 nm with the step of 25 nm.

The experimental samples with multilayer nanodots were based on dipolar coupled ultrathin Co layers with four, five, six and seven repetitions of the Pt/Co/Au trilayer (see Figure 1a). The Pt/Co/Au-based trilayer was chosen so that both dipole and DMI interactions influence the static micromagnetic state. All layers were sputtered in situ step by step on a Si substrate by DC magnetron sputtering at room temperature (base pressure was approximately 8 × 10^−5^ Pa; working gas was Ar at 0.175 Pa). The thicknesses of the deposited layers were defined by the rotation speed of the substrate over the magnetrons, and they were as follows: Pt = 1.5 nm, Co = 1.2 nm, Au = 1.5 nm. Then electron-beam lithography with a positive tone resist was used to define multiple arrays of dots with variable diameters varied from 150 to 525 nm. After the development, a 15 nm thick titanium layer was evaporated and lifted off to reveal Ti circle-shaped mask patterns. Finally, ion-beam etching was used to transfer the Ti-mask pattern into the Pt/Co/Au multilayer resulting in formation of stacked nanodots. The total height of the nanodots was 45, 52, 58 and 64 nm for samples with 4, 5, 6 and 7 repetitions of the Pt/Co/Au trilayer (see Figure 1b).

### 2.2. Measurements

Magnetic characterization of the presented sample was carried out using a vibrating sample magnetometer (VSM) with an in-plane magnetic field up to 2T. The obtained value of the magnetization saturation was about Ms = 1.1 × 10^6^ A/m. We expected that the Ms does not strongly depend on the number of trilayer repetitions. The measurement for 7 repetitions was performed, and this value of Ms was used to evaluate DMI.

The DMI strength was evaluated by the Brillouin light scattering (BLS) measurements in the Damon–Eshbach configuration [21,22,23]. By this system, one can measure the frequency shift Δ*f_SW_* between the counter-propagating spin waves at the specific value of the spin-wave wavenumber *k_SW_*. The value of Δ*f_SW_* corresponds to the shift between the Stokes and anti-Stokes frequencies, and then the value of DMI constant D can be directly estimated as:

(1) $$D=\frac{\Delta f_{SW}\pi M_{s}}{2\gamma k_{SW}}$$

where *γ* = 176 GHz/T is the gyromagnetic ratio. The measured values of frequency shift and DMI constant for the spin waves with the wavevector *k_SW_* = 0.011 nm^−1^ were calculated using Equation (1) and are summarized in Table 1.

The magnetic states of multilayered nanodots were investigated and induced by MFM with low-magnetic-moment tip (LM) and high-magnetic-moment tip (HM), respectively. The measurements were performed at room temperature with tip scanning speed of 5 μm/s. In both LM and HM scans, a standard two-pass MFM method was used. At first, sample topography was recorded in semi-contact mode (1st pass). During this scan, MFM tip was moving along the *x*-axis—from left to the right side of a dot array—then was returned back to the beginning of the same line. Within the second pass, the tip was lifted up (in z-direction) at a distance of 15 nm above the sample to lower Van der Waals forces and to image only magnetic forces. The tip’s motion was repeated according to sample topography. Next, the tip position was shifted along the *y*-axis by 15 nm to continue scanning on the next line. Note that in the two-pass MFM method, tip scans over each line four times.

All samples were firstly scanned by LM super-sharp probes (SSS-MFMR, NANOSENSORS^TM^, Neuchatel, Switzerland), with tip radius below 15 nm optimized for high-resolution MFM imaging. The spatial resolution of the LM probe was evaluated from fast Fourier transform analysis according to [24] and was estimated to have a value of 11 nm. The magnetic coating of these probes is characterized by a very low effective magnetic moment of 8 × 10^4^ A/m, which helps to minimize the invasive interaction between the tip and sample. The scans taken with LM probe revealed initial magnetic states in as-fabricated dots without affecting them by measurements. Next, HM probe with approximately four times higher magnetic moment (MESP, Bruker, Billerica, MA, USA) was magnetized in an external magnetic field in order to set it into a single-domain state. Subsequently, the same dot array was scanned by the HM probe whose stray field affected magnetization in dots while scanning. Finally, new magnetic states were measured by the LM probe again. Four identical dot arrays on each sample were scanned to check the reproducibility of the process.

### 2.3. Micromagnetic Simulations

**Figure 2:**
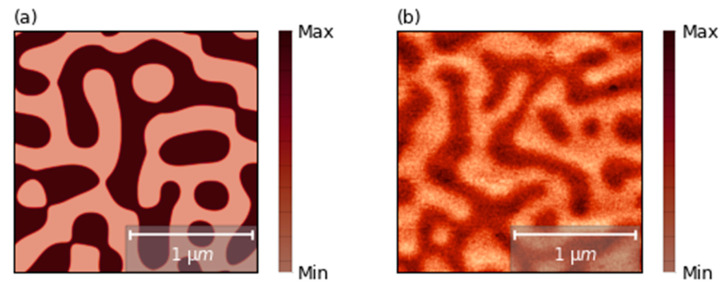
(**a**) The simulated domain distribution of the ultrathin films with seven repetitions of Pt/Co/Au with fitted exchange constant: A = 1.0 × 10^−11^ J/m. (**b**) MFM image of the domain distribution in ultrathin non-patterned Pt/Co/Au multilayer with seven repetitions.

The micromagnetic simulations of magnetic states in nanodots were performed using Boris spintronics solver [25]. This software uses a multilayered convolution algorithm [26], which allows one to calculate demagnetizing fields in magnetic multilayers with arbitrary spacing and thicknesses, without affecting computational performance. The simulations were performed using a uniformly discretized grid with a cell size of 1.5 nm × 1.5 nm × 1.2 nm. For the simulations, we employed following measured parameters: interfacial Dzyaloshinskii–Moriya interaction D = 1.0 mJ/m^2^, magnetization saturation Ms = 1.1 × 10^6^ A/m, a perpendicular magnetic anisotropy of Ku = 1.26 × 10^5^ J/m^3^ [10] and an exchange constant A = 1.0 × 10^−11^ J/m. In order to estimate the exchange constant strength, we measured and analyzed three different MFM scans of the non-patterned sample with seven repetitions (with the size 5 × 5 μm^2^, 10 × 10 μm^2^ and one with the size 20 × 20 μm^2^); one of them is presented in Figure 2b. We estimated the magnetic domain thickness in a multilayer by using 2D fast Fourier transformation (FFT) [27]. The micromagnetic simulations were performed for exchange ranging from 0.8 × 10^−11^ to 1.55 × 10^−11^ J/m with step 0.05 × 10^−11^ J/m. Similarly, FFT was used to estimate the domains’ width. The value of A = 1.0 × 10^−11^ J/m is the value that leads to the best correspondence between experiment and simulations. The simulated domain corresponding to this value is presented in Figure 2a.

## 3. Results and Discussion

### 3.1. Inducing Single-Domain State

**Figure 3:**
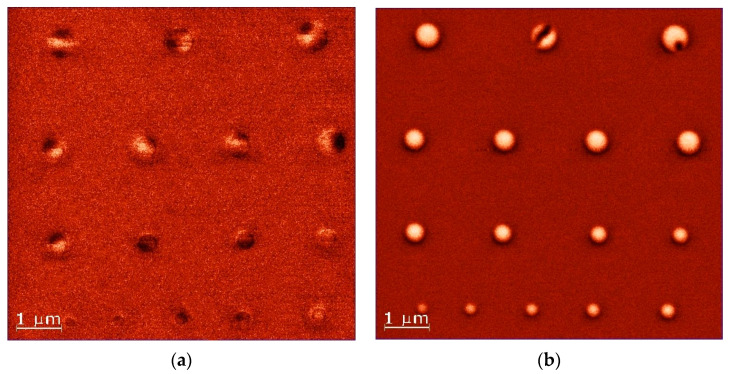
The figure shows the distribution of magnetization in nanodots taken by LM tip, which was done before (**a**) and after (**b**) scan by HM tip. (**a**) shows complex domain structures, including horseshoe domains (i.e., 525, 500, 475, 375 nm), while (**b**) shows single-domain states in all dots with a diameter smaller than 500 nm.

We compared the results of LM MFM measurements of magnetic states before and after HM tip scan (Figure 3a,b, respectively). We found that the MFM probe influence on magnetic states in dots was very similar for all samples. As the MFM scans of samples with 7 repetitions of Pt/Co/Au demonstrated the highest quality, we decided to present only the results for that sample. Both LM measurements and HM tip scan were performed with two-pass techniques as described in the methods section. We observed that the magnetic states were strongly influenced by the HM tip scan. A single-domain state was induced in almost all of the disks. Only in the disk with the largest diameter (500 and 525 nm), the magnetic domain remained, but it was different than in the first scan. This might indicate the limit of the thermal stability of the single-domain state. The same procedure (LM measurements, HM scan and LM measurements) was repeated for four arrays with a disk diameter of 150–525 nm for four different samples providing qualitatively the same results. 

We performed a numerical simulation to gain more insight into the process of remagnetization of domain structures to a single-domain state. We approximated the magnetic field profile induced from the tip as a Gaussian function (homogeneous through the thickness of the sample with 50 nm full width at half maximum). In the next part of our manuscript, the magnetic field induced by the tip is called the tip field. 

Due to the fact that a local MFM tip was able to remagnetize magnetic samples to a single-domain state in large dots (up to 475 nm), we assume that the local pinning of the sample plays an important role in the process of inducing a single-domain state. To mimic the polycrystalline nature of the sample, a Voronoi tessellation was added in the micromagnetic simulations where, to each grain of 6 nm in size, a slightly different perpendicular magnetic anisotropy was assigned. The anisotropy value was chosen randomly from a normal distribution centered around a mean value of Ku = 1.26 × 10^5^ J/m^3^ with a standard deviation of 35% [10,13]. 

In the simulations, we reproduced the MFM scan path by scanning each horizontal line twice—left to right and right to left—and then moving to the next line, bottom to top. The horizontal and vertical step was set to 15 nm. To reproduce this procedure in simulations, we relaxed magnetization for each position of the tip field. A characteristic time scale over which a perturbed magnetic domain will relax is in the range of a few nanoseconds, much shorter than the scanning time of a single point (approx. 3 ms). We performed simulations for various tip field amplitudes, from 100 to 1500 mT, to find the minimal strength and size of the magnetic field induced by the MFM tip and verify the necessity of local pinning sites in the sample. We found that when the magnetic field is weak (below 200 mT), the influence of the tip field is not sufficient to influence the domain structure, neither by remagnetization nor moving of domains. The tip field with a strength of 200 mT is strong enough to move and annihilate domains at the boundaries. However, it is not sufficient to re-magnetize the large-domain or single-domain state with opposite polarity. A field of 1150 mT is needed to re-magnetize a domain of any size (also a single-domain state). 

**Figure 4:**
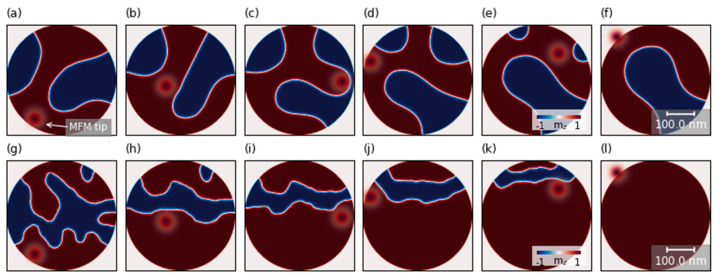
Simulated images of the z-component of the magnetic configuration evolution without pinning centers (**a**–**f**) and with the pinning centers (**g**–**l**) in nanodots dependent on position of the local magnetic field. The red circle represents the z-component of the magnetic field induced by the high-momentum tip. Simulations were performed for 400 nm disks with 7 repetitions of the stack with DMI = 1.0 mJ/m^2^. The amplitude of the local magnetic field was 350 mT at the center of the spot, and spot diameter was equal to 50 nm. The colors represent the orientation of the z-component magnetization.

The evolution of the exemplary random magnetic domains under the influence of the tip field with a strength value of 350 mT is represented in Figure 4. At first, we present a simulation without considering local pinning from the heterogeneity of the sample (a)–(f). In these simulations, the simulated scanning of the MFM tip leads to domain movement and the formation of a horseshoe state (shown in Figure 4a–f) or multi-domain states. For a particular initial state (Figure 4a), we observed that the MFM tip expands locally the domain with the polarity of the tip (red domain). When the tip scans the lower part of the disk, the red domain expands when the tip is above the disk and shrinks back when the tip is outside of the disk. When the tip scans near the central part of the disk, expansion of the red domain leads to division of the blue and the formation of three separate smaller domains (Figure 4b–d). In subsequent scan steps, when the tip is scanning the upper part of the disk, the remaining smaller domains are annihilated, creating a horseshoe state (Figure 4e–f).

Taking into account the non-uniformities of the sample (g)–(l) allows the creation of strong pinning centers for the domain, and it prevents shrinking of the red domain when the tip is outside of the disk. Therefore the domains are successively moved by the tip field during the scan and are annihilated at the upper boundary. Domain behavior at these selected fields observed in the simulations may differ in relation to experimental measurements, taking into account values of magnetic material parameters or heterogeneity of magnetic parameters [28,29,30]. The results of these simulations confirm our hypothesis that the experimentally produced samples exhibited strong granularity leading to strong local pinning sites.

### 3.2. Inducing Skyrmion

Next, we performed numerical simulations to demonstrate the potential of MFM-tip-induced formation of the isolated skyrmion in the dots with uniform properties inside the area of the dot (without the pinning centers) and non-uniformities at the dot boundaries. The origin of the boundary pinning might be due to non-uniformities at the boundaries [31,32], thickness non-uniformities [33] or oxidation at the edges [34,35]. The pinning centers at the boundaries might be also introduced intentionally during the fabrication process as local defects. In order to take into account the pinning effects at the dot edges, we defined an outer ring of 10 nm in width. In this region, we added a Voronoi tessellation where, in each 6 nm grain, a different value of the perpendicular magnetic anisotropy was assigned. The value of anisotropy in these grains was chosen with a normal distribution centered around a nominal value with a standard deviation of 35%. 

**Figure 5:**
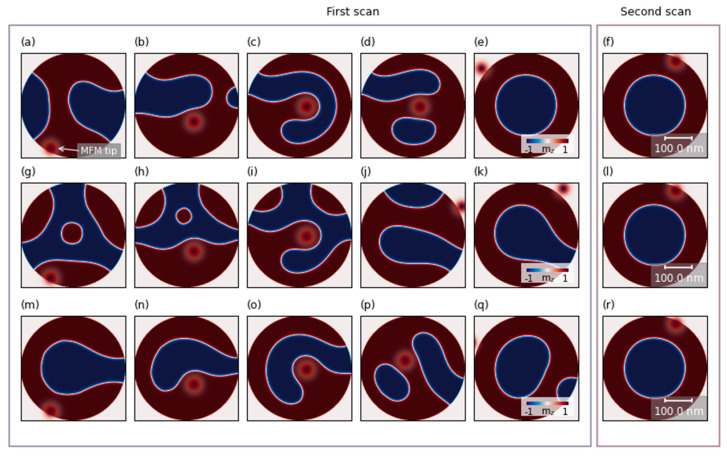
Simulated images of the magnetic configuration evolution to the skyrmion state (**a**–**e**), horseshoe state (**g**–**k**) and skyrmion and small domain state (**m**–**q**) depending on initial magnetization configuration (**a**,**g**,**m**). The right column of the left frame shows the simulated final state after the first scan. The right frame shows the simulation result after the second scan of the previous final state (**f**,**l**,**r**). The red circle represents the z-component of the magnetic field induced by the high-momentum tip. The red and blue colors correspond to the positive and negative out-of-plane components of the magnetization, respectively.

In the case when the value of the tip field was sufficiently strong to move the domains (over 200 mT), the MFM scan nucleated the skyrmion. We observed that the process of domain shifting and domain formation depends on the initial state of magnetization in the nanodot. Exemplary evolution of the magnetization in 400 nm nanodots during the HM scan is shown in Figure 5 for three different initial states. The polarization of the tip field in these simulations corresponds to the polarization of the domain with positive polarization (marked with red color). Therefore the tip field will lead to expansion of the domains with positive polarization. The HM tip scanning process shifts or annihilates the domains by pushing or pulling smaller domains away from the edge of the nanodot. Often this process leads to smaller domains merging into one. In Figure 5b, it is shown how the tip pushes up the domain with negative polarization (blue domain) as the scanning proceeds from bottom to top. The pinning centers prevent moving back of the domain at the edges, but it can move freely in the middle of the disk. This leads to an intermediate state, with a large domain of positive polarity filling the bottom half of the nanodot’s edges and a mixed domain pattern in the rest of the disk (Figure 5b). When the tip field moves away from the sample, the domain with negative polarity fills the empty space in the middle of the nanodot, which forms the horseshoe state (see Figure 5c). The horseshoe state is one of the most common realizations of this process due to its low energy level [36], in which only a small portion of the domain is pinned to the edge of the nanodot. Next, the magnetic tip continues the scan and splits the horseshoe domain into a skyrmion and a remaining domain (see Figure 5d). The result is a skyrmion with opposite polarity to the magnetic tip (Figure 5e), which was formed due to the increase of surface tension inside the horseshoe domain wall [37], caused by the repulsion of the domain by the tip-induced field. This process of skyrmion formation by cutting off a fragment of the domain wall is similar to the process of domain wall detachment by electric current pulse [37]. The topological charge was calculated across the thickness of the dot. For all seven layers, we observed that the skyrmion topological number calculated separately for each layer is close to 1. We also studied numerically whether the scanning HM-MFM can destroy the skyrmion. We performed a second MFM and did not observe any changes in the magnetic state when the skyrmion was in an initial state (see Figure 5f). As the field moved, the skyrmion was pushed from its central position, contracting or stretching around the tip field. These results show that repeated HM-momentum tip scanning can lead to an increase in skyrmion formation probability.

Depending on the initial magnetization state, the first MFM scan does not always lead to skyrmion formation. We performed simulations for 20 random initial states (see Figure 5g–k for the evolution of the magnetic scan during the MFM scan when another labyrinth domain structure was an initial state and Figure 5m–q when horseshoe was an initial state). Most often, we observed induction of a horseshoe state (see Figure 5k) or a skyrmion and small domains near the edge (Figure 5q). After the second scan, we obtained a skyrmion state or skyrmion and a small-domain state in the vast majority of simulations. The results of the second HM-MFM scan are shown in the right panel of Figure 5f,l,r, where magnetization states from Figure 5e,k,q are taken as an initial state for each case, respectively. We did not observe horseshoe state disintegration during the second scan for other multi-domain states. 

## 4. Conclusions

In summary, we demonstrated that the local magnetic field induced by the MFM tip can influence the magnetic state of a nanodisk. The experimentally measured disks were remagnetized from the domain state to the single-domain state. The numerical simulations indicated the important role of pinning centers in that process. Further, with numerical simulations, we showed that control of pinning centers within the area of the dot and at the boundary of the dot can lead to a reproducible method of skyrmion nucleation in the nanodots. We demonstrated the evolution of remagnetization from the domain state to skyrmion and identified the important role of the intermediate horseshoe state in that process. 

## Figures and Tables

**Table 1:** The measured value of frequency shift and calculated DMI constant.

Sample	Δ*f_SW_*, GHz	D, mJ/m^2^
(Pt(1.5)/Co(1.2)/Au(1.5))_×4_	1.12 ± 0.1	1.0 ± 0.1
(Pt(1.5)/Co(1.2)/Au(1.5))_×5_	1.16 ± 0.1	1.04 ± 0.1
(Pt(1.5)/Co(1.2)/Au(1.5))_×6_	0.89 ± 0.1	0.79 ± 0.1
(Pt(1.5)/Co(1.2)/Au(1.5))_×7_	1.12 ± 0.1	1.0 ± 0.1

## Data Availability

The data that support the results of this study are available from the corresponding author upon reasonable request.
